# The value of MR-based radiomics in identifying residual disease in patients with carcinoma in situ after cervical conization

**DOI:** 10.1038/s41598-020-76853-1

**Published:** 2020-11-16

**Authors:** Mengfan Song, Jing Lin, Fuzhen Song, Dan Wu, Zhaoxia Qian

**Affiliations:** 1 Department of Obstetrics and Gynecology, International Peace Maternity and Child Health Hospital, School of Medicine, Shanghai Jiaotong University , Shanghai, 200030 China; 2 Department of Radiology, International Peace Maternity and Child Health Hospital, School of Medicine, Shanghai Jiaotong University , Shanghai, 200030 China; 3 Department of Cervical Disease, International Peace Maternity and Child Health Hospital, School of Medicine, Shanghai Jiaotong University School of Medicine, Shanghai, 200030 China; 4 Shanghai Key Laboratory of Embryo Original Diseases, Shanghai, 200030 China

**Keywords:** Magnetic resonance imaging, Disease-free survival, Cervical cancer

## Abstract

Carcinoma in situ (CIS) of the uterine cervix is a precursor to cervical carcinoma. However, hysterectomy can be avoided in patients who can be treated by cone biopsy. Previous studies have shown that imaging-based approaches allow for the noninvasive visualization of cervical cancer, and radiomics has high accuracy in classifying cancer and predicting treatment outcome for different cancer types. To develop a magnetic resonance (MR)-based radiomics model for identifying residual disease in patients with CIS after cervical conization. Patients who had CIS after conization and finally underwent hysterectomy were collected to comprise a database to establish an imaging model for predicting the residual status after conization. Then, patients who opted for uterine preservation were classified as high-risk or low-risk patients according to the model. The disease-free survival was compared between the different risk groups using the Kaplan–Meier curve. The model built with the Boruta features outperformed the random forest model. Further validation with patients with uterine preservation showed that the patients classified as high risk were more likely to have tumor recurrence/residual disease in the follow-up period. In conclusion, radiomics can be used to identify residual disease in patients with CIS after cervical conization and could have the potential to predict recurrence in patients who opt for uterine preservation.

## Introduction

Carcinoma in situ (CIS) of the uterine cervix is a precursor to cervical carcinoma^1^. A cone biopsy procedure, including cold knife cone (CKC) biopsy or a loop electrosurgical excision procedure (LEEP), is mandatory to exclude invasive disease, and in some cases, patients can even be treated by cone biopsy^2^. This fact shows that hysterectomy can be avoided in some patients. However, compared to patients without residual disease, patients with residual disease have a significantly higher risk of recurrence, and recurrence occurs earlier^3–6^. Residual disease can be identified with repeat colposcopy and biopsy through endocervical curettage (ECC) or excisional procedures. However, these procedures are invasive, and patients who undergo a repeat surgery can experience difficulties with fertility, both in becoming pregnant or maintaining their pregnancy, if desired^7^.

Previous studies have shown that imaging-based approaches allow for the noninvasive visualization of cervical cancer, and magnetic resonance imaging (MRI)-based approaches have shown potential in detecting the residual status of many other diseases^8–10^. Although promising, the conventional MR approach based on morphological evaluations to predict residual status has not been well recognized since the summarized morphological features and consistency of this model would be a challenge for using this approach on a large scale. Recently, radiomics has been recognized as an emerging technique that converts medical images into high-dimensional mineable data by means of feature engineering and machine learning techniques^11–13^. Recent advances in medical imaging (including radiomics) allow for high-throughput extraction of information imaging features. These features help us quantify the temporal heterogeneity at different levels of genes, proteins, cells, micro-environments, tissues and organs. Although it limits the use of molecular analysis for invasive biopsies, radiomics provides significant room for medical imaging, which non-invasively acquires intratumoural heterogeneity. Some of the previous studies^14,15^ have demonstrated that radiomics features offer information about the cancer phenotype and the tumor microenvironment. Radiomics has been reported to have high accuracy in classifying cancer and predicting treatment outcomes for different cancer types^16^. However, until now, no study has assessed radiomics features to evaluate the residual status of CIS after cervical conization.

Thus, the purpose of this study was to investigate the performance of an MR-based radiomics model in evaluating the residual status of CIS after conization.

## Results

### Patient characteristics

The mean (SD, range) age of the patients was 47.9 (14.3, 21–76) years old for the training group and 49.3 years old (12.9, 25–72) for the test group. The mean (SD, range) interval between the 2 operations was 6 (3.3) weeks for the training group and 6 (3.5) weeks for the test group. The mean time from conization to MRI was 2.6 weeks (ranging from 16 to 43 days) for the training group and 2.4 weeks for the test group (ranging from 14 to 35 days ). In the training group and test group, 24 and 22 patients had positive lesions at the margins of all quadrants, while residual disease was identified in 18 and 17 patients, respectively. The groups with or without residual disease were similar with respect to age and the interval between the training and test procedures (all p values > 0.5). For the validation group, which included patients who chose uterine preservation, the mean (SD, range) age of the patients was 39.9 (19.3, 19–53) years old, none of the patients had a positive margin, and 3 patients had residual invasive carcinoma.

### Establishment of the imaging model

**Figure 1:**
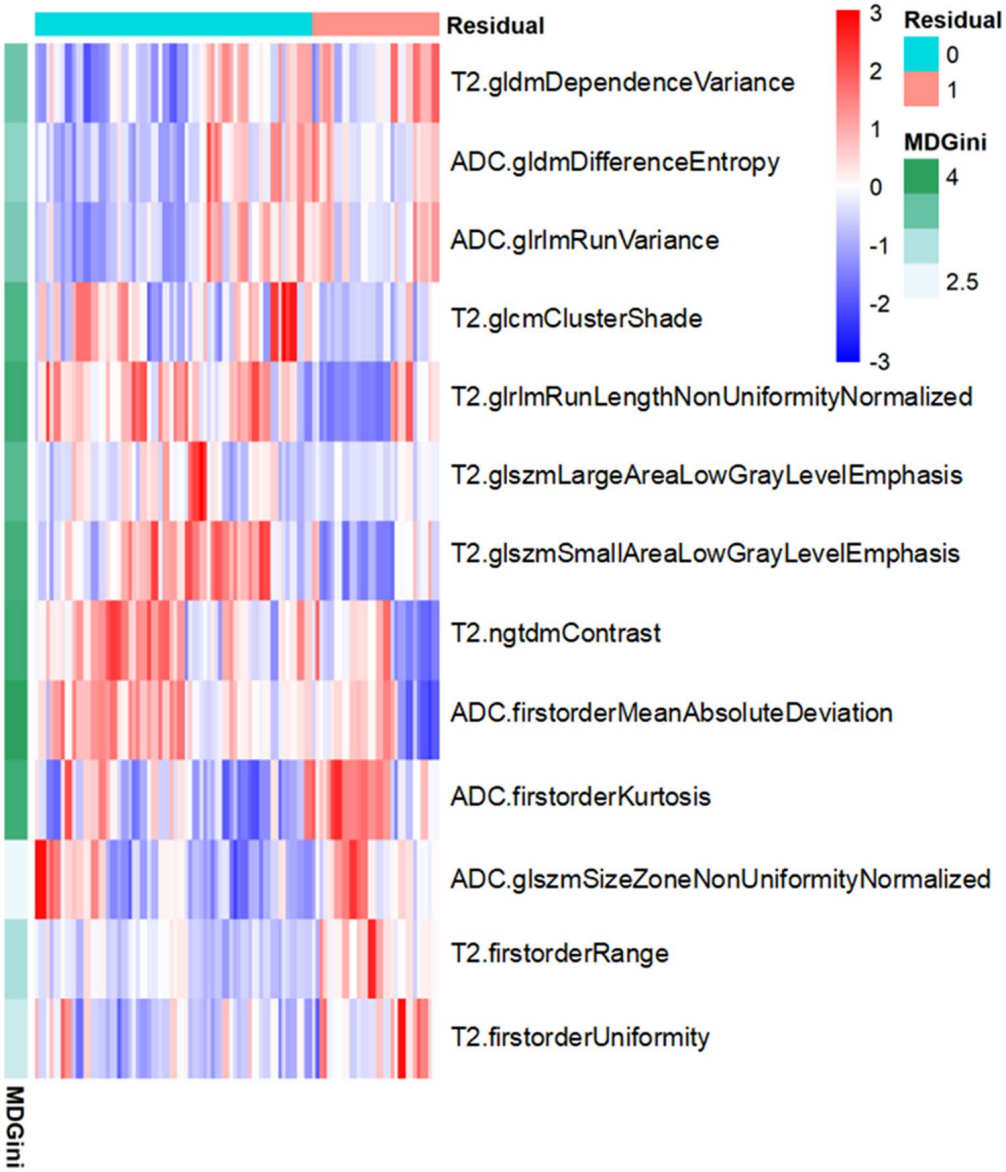
Heatmap of the normalized feature value distribution of the 13 all-relevant features to differentiate between residual and nonresidual disease.

**Figure 2:**
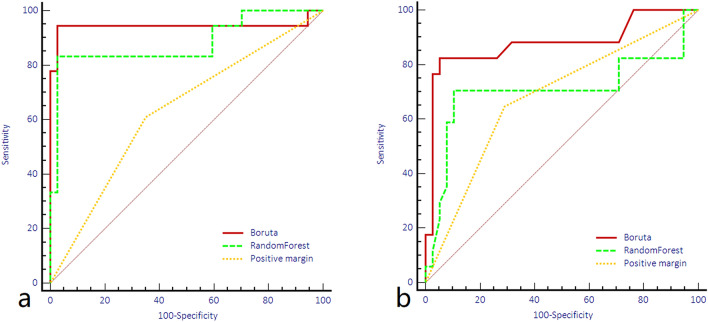
ROC curves of the random forest and all-relevant models for identifying residual disease in the training (**a**) and test (**b**) cohort.

Based on the MR images, 156 features could be extracted for a single sequence. After the intra- and interobserver repeatability tests, 42 T2WI features and 59 DWI features remained, and 13 all-relevant features were selected by the Boruta method. The feature selection results are summarized in Table 1, and a heatmap showing the value distribution of the features selected by the Boruta method between the residual and nonresidual groups is shown in Fig. 1. The ROC curves of the radiomics model with or without the Boruta method for differentiating residual disease from nonresidual disease in the test cohort are shown in Fig. 2. The performance is summarized in Table 2. The model built with the Boruta features achieved an AUC of 0.889 and accuracy of 87.3% in the test cohort and outperformed the random forest model, which had an AUC of 0.701 and accuracy of 72.1%. The DeLong analysis found a significant difference between the AUCs of these two models (p = 0.039). Moreover, the radiomics models had better performance than the positive margins for differentiating between residual and nonresidual disease (p = 0.004).

**Table 1:** A summary of the extracted and selected radiomics features.

	Extracted radiomics features		All-relevant features (Boruta)	
	T2WI	ADC	T2WI	ADC
First order features	“InterquartileRang”,“Skewness”,“Uniformity”,“Median”,“Energy”,“RobustMeanAbsoluteDeviation”,“MeanAbsoluteDeviation”,“Maximum”,“RootMeanSquared”,“90Percentile”,“Minimum”,“Range”,“Variance”,“10Percentile”,“Kurtosis”,“Mean”		Range, Uniformity	MeanAbsoluteDeviation, Kurtosis
Shape features	“Maximum3DDiameter”,“Maximum2DDiameterSlice”,“Sphericity”,“MinorAxis”,“VolumeRatio”,“Volume”,“MajorAxis”,“SurfaceArea”,“Flatness”,“LeastAxis”,“Maximum2DDiameterColumn”,“Maximum2DDiameterRow”			
GLCM/GLDM features	“GrayLevelVariance”,“HighGrayLevelEmphasis”,“DependenceEntropy”,“DependenceNonUniformity”,“GrayLevelNonUniformity”,“SmallDependenceEmphasis”,“SmallDependence”,“HighGrayLevelEmphasis”,“LargeDependenceEmphasis”,“LargeDependenceLowGrayLevelEmphasis”,“DependenceVariance”,“DependenceHighGrayLevelEmphasis”,“LowGrayLevelEmphasis”,“JointAverage”,“SumAverage”,“ClusterShade”,“Idmn”,“JointEnergy”,“Contrast”,“DifferenceEntropy”,“InverseVariance”,“DifferenceVariance”,“Idn”,“Idm”,“Correlation”,“Autocorrelation”,“SumSquares”,“ClusterProminence”,“DifferenceAverage”,“ClusterTendency”		ClusterShade, DependenceVariance	DifferenceEntropy
GLRLM features	“ShortRunLowGrayLevelEmphasis”,“GrayLevelVariance”,“LowGrayLevelRunEmphasis”,“GrayLevelNonUniformityNormalized”,“RunVariance”,“GrayLevelNonUniformity”,“LongRunEmphasis”,“ShortRunHighGrayLevelEmphasis”,“RunLengthNonUniformity”,“ShortRunEmphasis”,“LongRunHighGrayLevelEmphasis”,“RunPercentage”,“LongRunLowGrayLevelEmphasis”,“HighGrayLevelRunEmphasis”,“RunLengthNonUniformityNormalized”		RunLengthNonUniformityNormalized	RunVariance
GLSZM features	“GrayLevelVariance”,“ZoneVariance”,“GrayLevelNonUniformityNormalized”,“SizeZoneNonUniformityNormalized”,“SizeZoneNonUniformity”,“GrayLevelNonUniformity”,“SmallAreaHighGrayLevelEmphasis”,“ZonePercentage”,“LargeAreaLowGrayLevelEmphasis”,“LargeAreaHighGrayLevelEmphasis”,“HighGrayLevelZoneEmphasis”,“SmallAreaEmphasis”,“LowGrayLevelZoneEmphasis”,“ZoneEntropy”,“SmallAreaLowGrayLevelEmphasis”		SmallAreaHighGrayLevelEmphasis, LargeAreaLowGrayLevelEmphasis	SizeZoneNonUniformityNormalized
NGTDM features	“Coarseness, Complexity”,“Strength”,“Contrast”,“Busyness”		Contrast

**Table 2:** Internal validation of the performances of RandomForest model, Boruta model for differentiating residual and nonresidual disease.

	Training group			Test group		
	RF	Boruta	PM	RF	Boruta	PM
AUC	0.899	0.959	0.630	0.701	0.889	0.679
Accuracy	0.891	0.963	0.636	0.721	0.873	0.691
Sensitive	0.722	0.944	0.611	0.706	0.824	0.647
Specificity	0.973	0.973	0.649	0.749	0.895	0.711
PPV	0.929	0.944	0.572	0.667	0.778	0.591
NPV	0.878	0.973	0.734	0.805	0.919	0.758

*RF* RandomForest, *PM* positive margin.

### Validation of the imaging model

**Figure 3:**
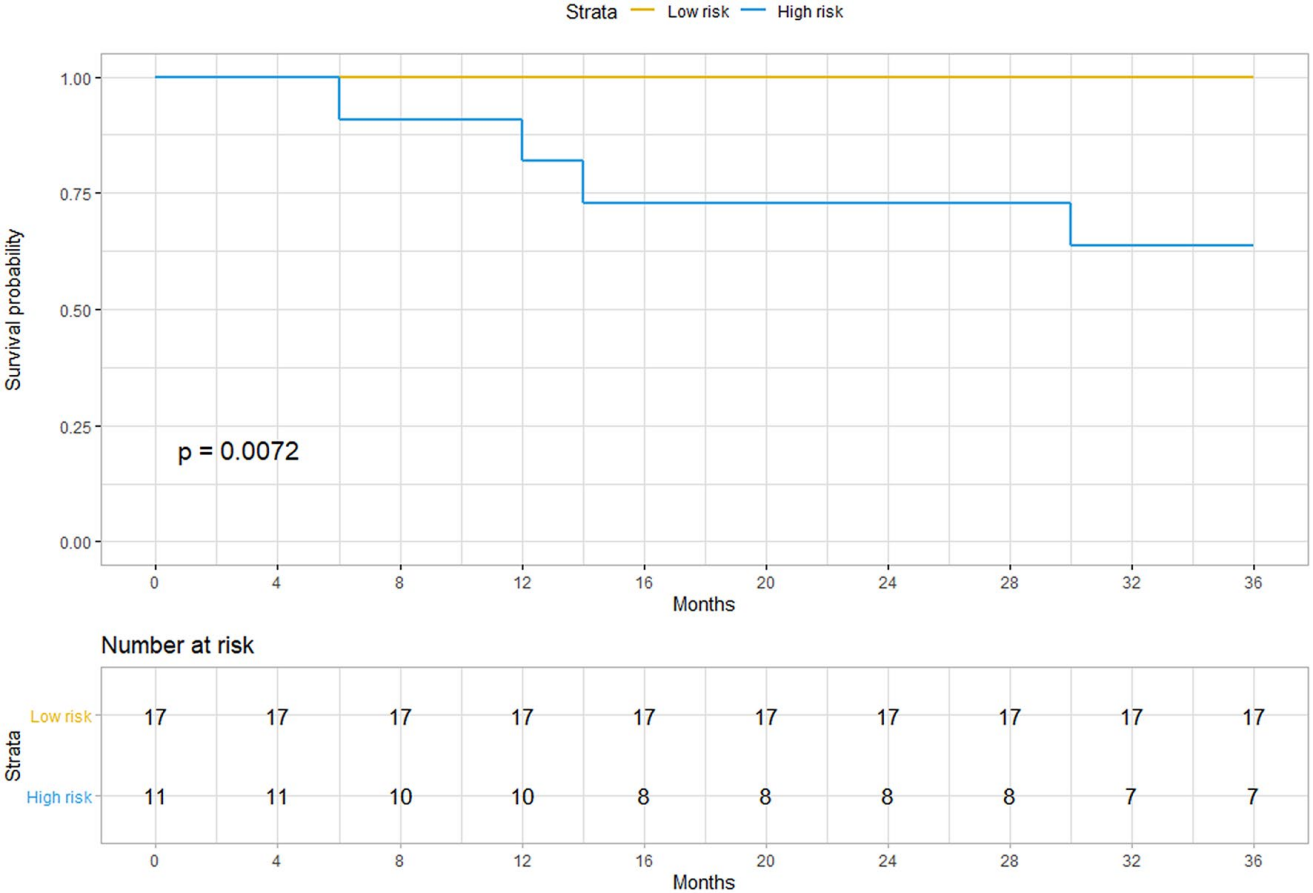
The Kaplan–Meier curve of the radiomics model for identifying high- and low-risk patients in the validation group.

The imaging model was further validated in patients who did not undergo hysterectomy. Among 28 patients, 11 were classified as high risk, and 17 were classified as low risk, according to the imaging model. One patient in the low-risk group was pregnant in the follow-up period. Four patients were found to have recurrence/residual disease in the follow-up period, and all of these patients were in the high-risk group. The Kaplan–Meier curve is shown in Fig. 3, and DFS between the high-risk and low-risk groups was significantly different (p = 0.007).

## Discussion

Since using traditional MRI to evaluate residual status is insufficient, very little MR research has reported reliable evaluations of residual disease after conization, which could be because the level of information captured by human vision is inadequate to characterize disease, or the information captured is not sufficient for a diagnosis. Thus, a high-throughput data mining method to extract features from images might improve the diagnostic performance of MRI. In our study, we extracted 156 features derived from 3D tumur regions in MR images with the goal of enhancing the reproducibility and usefulness of radiomics models^1,7^. Preprocessing, including image normalization and intra- and interobserver reproducibility tests, was performed before modelling. We used feature selection processes to screen the radiomics metrics and form the model. Finally, we developed a radiomics model that can be used to predict residual disease after conization in patients with CIS with relatively high accuracy. The model can be used to identify patients at high risk for residual disease, while patients with low risk might not need a radical treatment plan in the follow-up period. The results of our study showed that, although it might not be easily detected by radiologists, residual disease did exist on MR images, which could be evaluated by the radiomics imaging features. Although the radiomics features are often linked with heterogenicity, complexity, and entropy inside the tumur^14–18^, we believe that residual disease could also be considered a variant of normal tissue or inflammation and could certainly be reflected by the radiomics features.

In addition to feature extraction, using accurate methods is also important for the performance of the final radiomics model. Recently, the Boruta method and minimum redundancy maximum relevance ensemble (mRMRe) have been successfully used as feature selection tools in radiomics^19,20^. A previous study demonstrated that all-relevant features are not necessarily features with maximum relevance; maximizing the mutual information relevance might not always maximize the classification accuracy^2,1^. Compared with mRMRe, the all-relevant features selected by Boruta had better predictive performance. Thus, we chose the Boruta method to select the features for our imaging model; compared with the conventional random forest method, using the Boruta method to select all-relevant features led to a better performance in our study.

Thus far, the management of patients with positive surgical margins after conization has been controversial^22,23^. The persistence and recurrence rates among these patients in follow-up are higher than those among patients with negative margins^2,4^. Positive margins might be associated with invasive carcinoma. However, the results have varied between studies. Kietpeerakool et al.^2,5^ noted that the incidence of cervical cancer in patients with LEEP-positive margins was 0.9–9.6%. In our study, we found accuracy of 67.9% using the positive margins, which was significantly inferior to that of the radiomics model. We consider that an association between positive margins and residual disease might exist. However, the emergence and evaluation of this feature from actual clinical practice are unreliable and uncertain, and the imaging model might present a more robust and more comprehensive way of predicting residual disease after conization.

Residual disease is also an important clinical factor^26^ and can be associated with the DFS of patients. However, the safety of uterine preservation in patients with CIS still must be evaluated. Thus, we also retrospectively validated the radiomics model in a group of patients who did not undergo hysterectomy and showed that uterine preservation is safe for low-risk patients. Although the patients who did not undergo hysterectomy were younger than the mean age of the entire cohort, which might have led to some bias in the evaluation, in our opinion, these results should reflect to the real-world clinical situation since younger patients are more likely to opt for uterine preservation. Our study showed that all of the patients who had recurrence after conization were identified as high-risk patients, and at the end of the follow-up period, none of the patients in the low-risk group developed recurrence. Thus, low-risk patients can safely avoid hysterectomy. Patients in different risk categories could consider different follow-up strategies. Certainly, further studies are needed to improve on the retrospective design of this study and expand upon our limited number of cases. Moreover, a longer follow-up duration is needed.

In our study, we developed a semi-automated procedure before feature extraction. The volume of the uterine area and the conization margins must be identified by the radiologist; although we evaluated the inter- and intraobserver agreement to include robust features to build the radiomics model, the procedure could be further improved if all of the segmentation steps for the VOI could be performed automatically, which is an area that has already been explored in other organs^27–29^. Moreover, we used 5 mm as the radius of outward corrosion from the LEEP margin since we assumed that most of the remaining lesions are within this range, and we appeared to be successful in the application.

The main limitations of our study are the limited number of patients with uterine preservation and the retrospective design. The criteria for uterine preservation in patients with CIS remain controversial, and uterine preservation is not the first choice in routine clinical practice. The true value of the radiomics model for patients with CIS and uterine preservation must be further validated. Moreover, the robustness of the model could be improved if the segmentation procedure could be fully automated. Finally, according to international community radiomics standardization, phantom reference for MR is not well established, so test–retest variability should be applied on phantom images to complete stability analysis in the future.

## Conclusion

In conclusion, the present results showed that the radiomics model could be used to predict residual disease after conization and could have the potential to predict recurrence in patients who opt for uterine preservation. However, as mentioned above, more investigations with better designs are needed to further validate the present findings.

## Methods and materials

The records of patients with a diagnosis of CIS proven by conization and who underwent an MR scan between March 2013 and March 2016 in our hospital were retrospectively reviewed. A total of 110 patients were included to form the database in the present study. Clinical and pathological variables, including age, parity, menopausal status, conization method, cone base area and depth, endocervical margin and glandular involvement, endocervical involvement based on ECC, and the number of quadrants with positive margins, were collected. The study was approved by Institutional Review Board of International Peace Maternity and Child Health Hospital, and written consent was obtained from all of the patients. All experiments were performed in accordance with relevant guidelines and regulations.

The study was retrospectively designed and performed in two stages. First, from the database, we collected patients who ultimately underwent hysterectomy to form a cohort and established an imaging model to predict the residual status after conization. Patients were randomly assigned to training and testing groups at a ratio of 1:1, and the performance of the imaging model was compared with the pathological positive margins in this stage. In the second stage, patients who opted for uterine preservation were included, and all of the patients were classified as high-risk or low-risk patients according to the imaging results. In this stage, the imaging model established in the first stage was used to classify the risk categories in patients with uterus preservation, and the performance of the model was evaluated. Patients with abnormal colposcopy or high-grade squamous intraepithelial lesion smear results in the follow-up procedure were subjected to repeat ECC, and the presence of histologically confirmed cervical intraepithelial neoplasia grade 2 or 3 (CIN2/3) or higher was considered residual or recurrent disease^30^. All of the patients were followed up for 36 months.

### MRI protocol

All scans were performed using a 1.5-T MR scanner (Aera, SIEMENS, Erlangen, Germany) with the patient in the supine position. The following sequences were used to acquire images, from which features were extracted for the radiomics model: axial T2-weighted imaging (T2WI) (repetition time/echo time = 4500 ms/80 ms, slice thickness = 6 mm, gap = 1 mm, field of view (FOV) = 320 * 240, flip angle = 160°, number of excitation = 2, with fat saturation), and axial diffusion-weighted imaging (DWI) (repetition time/echo time = 5200 ms/80 ms, field of view = 250 * 200, slice thickness = 6 mm, gap = 1 mm, flip angle = 90°, number of excitation = 6, b-value = 0, 800). The apparent diffusion coefficient (ADC) value was derived according to the following equation:

$$S\left( b \right) = S_{0} \exp ( - bADC)$$

where S(b) and S(b0) represent the signal intensity of a certain voxel in the presence and absence of diffusion sensitization, respectively.

### Area segmentation and radiomics feature extraction

**Figure 4:**
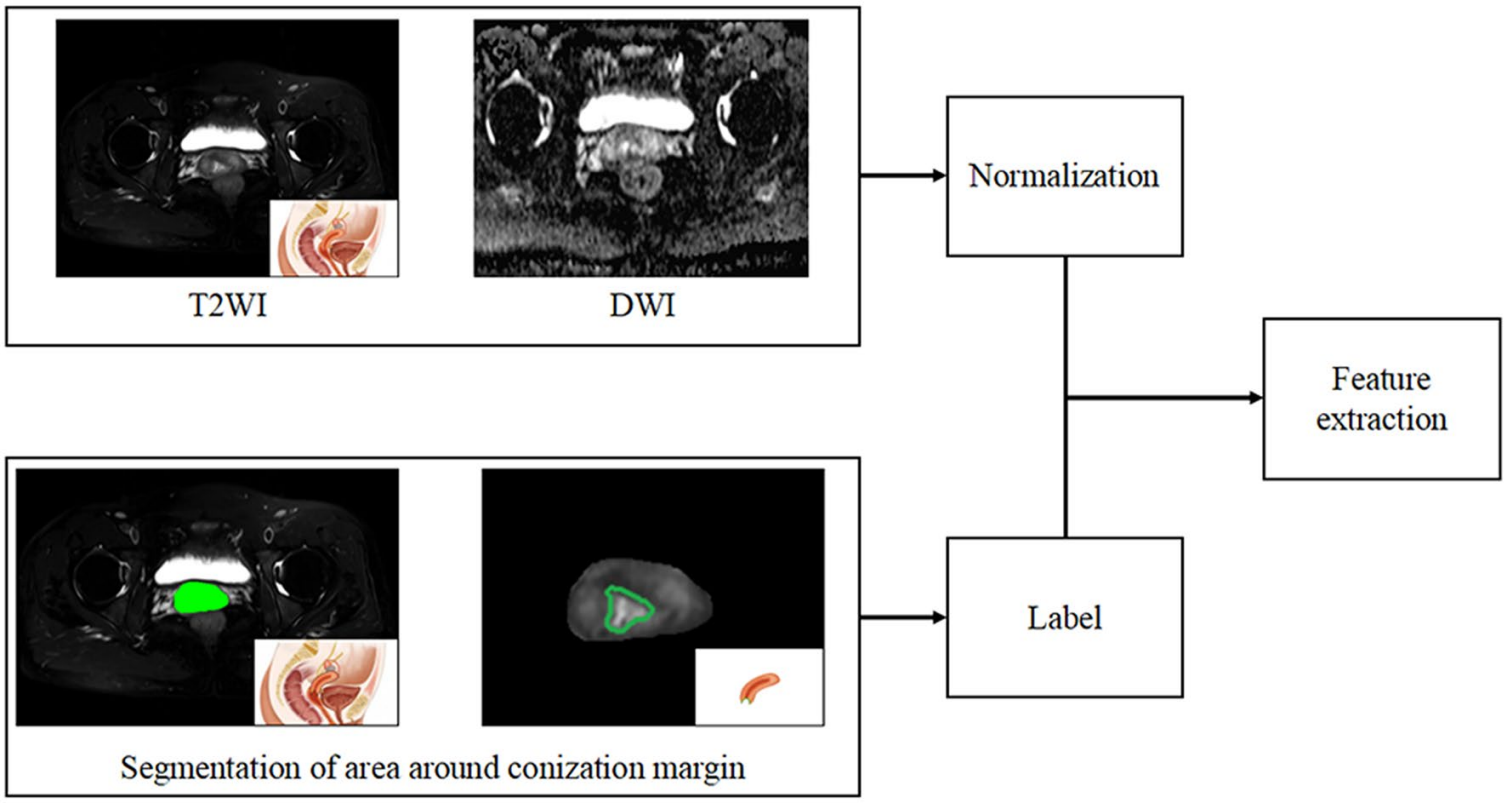
The flowchart of segmentation procedure.

This normalization approach was used according to a previous study^31^, Three-dimensional volumes of interest (VOIs) of tumour contours were manually delineated slice-by-slice by the radiologist (D.W., with more than 10 years of experience in pelvic imaging) using the ITK-SNAP software(version 3.2, http://www.itksnap.org), and VOIs were first drawn to segment the uterus on the T2W images to ensure the following segmentation area was within the uterus; then, based on the segmented uterine area, the cornization margin was delineated. Image erosion was applied to the binary segmentation mask for each cornization margin using a disk with a defined pixel radius, which was then eroded (disk radius of 3 pixels) to generate the VOI under the cornization margin for further feature extraction. The VOI delineated on the T2W image was also applied to the ADC maps. The segmentation procedure is shown in Fig. 4. For each segmented 3D volume, we extracted quantitative texture features from each phase using a program developed in house. The texture features describe the high-order spatial distributions of intensities within the VOIs. Fifty-two texture features were extracted from each sequence using several different methods, including the grey level co-occurrence matrix (GLCM), grey level run length matrix (GLRLM), grey level size zone matrix (GLSZM), and neighbourhood grey-tone difference matrix (NGTDM). A detailed calculation of the texture features can be found in^15,17,31^. For each VOI, 156 features were extracted from the MR image. To find robust features against the intra- and interobserver delineation variations, the delineation was repeated on 40 patients by the same radiologist (D.W.) to assess intraobserver reliability and by another clinician (M.S. with 4 years of experience in pelvic imaging) to assess interobserver reliability. Parameters were included only when the agreement was good.

### Statistical analysis

Intra- and interobserver agreement was analysed based on the intraclass correlation coefficient (ICC), and a parameter with an ICC greater than 0.75 was considered to have good agreement^3,2^. All of the classification models were trained on the training cohort and tested on the independent test cohort. Both feature subsets selected with or without the Boruta method were analysed. Multiple hypothesis correction was performed through a false discovery rate (FDR) adjustment using the Benjamini–Hochberg method^33^. The AUCs were statistically compared between different classifiers using the DeLong method. All of the indices were calculated for both the training and test cohorts. For the validation cohort, the high-risk and low-risk patients classified according to the imaging model were compared and evaluated using disease-free survival (DFS) with the Kaplan–Meier curve. The statistical analyses were performed with R software (version 2.9.1, http://www.r-project.org).

### Ethical approval and consent to participate

The study was approved by Institutional Review Board of International Peace Maternity and Child Health Hospital. Signed written informed consent was obtained from all participants.

## Abbreviations

MR: Magnetic resonance
CIS: Carcinoma-in-situ
DFS: Disease-free survival
VOI: Volumes of interest
GLCM: Gray level co-occurrence matrix
GLRLM: Gray level run length matrix
GLSZM: Gray level size zone matrix
NGTDM: Neighborhood gray-tone difference matrix
ICC: Intraclass correlation coefficient
mRMRe: Minimum redundancy maximum relevance ensemble
